# Supplementary material for: Zfhx3 Transcription Factor Represses the Expression of SCN5A Gene and Decreases Sodium Current Density (INa)
Source: Int J Mol Sci. 2021 Dec 2;22(23):13031. doi: 10.3390/ijms222313031 (PMC8657907; doi:10.3390/ijms222313031)
Supplement: Supplementary file 1 [file ijms-22-13031-s001.zip › ijms-1469250-supplementary.pdf]

Zfhx3 transcription factor represses the expression of *SCN5A* gene and decreases sodium current density (I_{Na})

SUPPLEMENTAL MATERIAL

Supplemental Figures

Supplemental Figure S1. V949 **(A),** M1260 **(B)** and Q2564 **(C)** aminoacids are highly conserved, as demonstrated by amino acid sequence alignments of Zfhx3 homologs in 15 different species. The red arrowhead and the vertical blue rectangle highlight the conservation of the corresponding residue and the horizontal red rectangle highlights predicted zinc-finger regions. "*" means that the residues in that column are identical in all the sequences included in the alignment, ":" indicates conservation between groups of strongly similar properties, and "." indicates conservation between groups of weakly similar properties.

A

p.V949I variant

```
Homo sapiens                    NDPSLKLFQCAVCNKFTTDNLDMLGLHMNVERSLSEDEWKAVMGDSYQCKLCRYNTQLKA 997
Pan troglodytes                 NDPSLKLFQCAVCNKFTTDNLDMLGLHMNVERSLSEDEWKAVMGDSYQCKLCRYNTQLKA 997
Vulpes vulpes                   NDPSLKLFQCAVCNKFTTDNLDMLGLHMNVERSLPEDEWKAVMGDSYQCKLCRYNTQLKA 996
Canis lupus familiaris          NDPSLKLFQCAVCNKFTTDNLDMLGLHMNVERSLPEDEWKAVMGDSYQCKLCRYNTQLKA 996
Equus caballus                  NDPSLKLFQCAVCNKFTTDNLDMLGLHMNVERSLPEDEWKAVMGDSYQCKLCRYNTQLKA 996
Trichechus manatus latirostris  NDPSLKLFQCAVCNKFTTDNLDMLGLHMNMERSLPEDEWKAVMGDSYQCKLCRYNTQLKA 992
Sus scrofa                      NDPSLKLFQCAVCNKFTTDNLDLLGLHMNVERSLPEDEWKAVMGDSYQCKLCRYNTQLKA 1002
Felis catus                     NDPSLKLFQCAVCNKFTTDNLDMLGLHMNVERSLPEDEWKAVMGDSYQCKLCRYNTQLKA 995
Gorilla gorilla                 NDPSLKLFQCAVCNKFTTDNLDMLGLHMNVERSLSEDEWKAVMGDSYQCKLCRYNTQLKA 999
Ursus arctos horribilis         NDPSLKLFQCAVCNKFTTDNLDMLGLHMNVERSLPEDEWKAVMGDSYQCKLCRYNTQLKA 995
Loxodonta africana              NDPSLKLFQCAVCNKFTTDNLDMLGLHMNMERSLPEDEWKAVMGDSYQCKLCRYNTQLKA 993
Bos taurus                      NDPSLKLFQCAVCNKFTTDTLDLLGLHMNVERSLPEEEWKAVMGDSYQCKLCRYNTQLKA 1006
Myotis lucifugus                NDPSLKLFQCAVCNKFTTDNLDLLGLHMNVERSLPEDEWKAVMGDSYQCKLCRYNTQLKA 993
Rattus norvegicus               NDPSLKLFQCAVCNKFTTDNLDMLGLHMNVERSLSEDEWKAVMGDSYQCKLCRYNTQLKA 996
Mus musculus                    NDPSLKLFQCAVCNKFTTDNLDMLGLHMNVERSLSEDEWKAVMGDSYQCKLCRYNTQLKA 998
                                *******************.**:******:**** *:***********************
```

B

p.M1260T variant

```
Homo sapiens                    KYSNADVNRLRVHAMTQHSVQPMLRCPLCQDMLNNKIHLQLHLTHLHSVAPDCVEKLIMT 1288
Pan troglodytes                 KYSNADVNRLRVHAMTQHSVQPMLRCPLCQDMLNNKIHLQLHLTHLHSVAPDCVEKLIMT 1288
Vulpes vulpes                   KYSNADVNRLRVHAMTQHSVQPMLRCPLCQDMLNNKIHLQLHLTHLHSVAPDCVEKLIMT 1296
Canis lupus familiaris          KYSNADVNRLRVHAMTQHSVQPMLRCPLCQDMLNNKIHLQLHLTHLHSVAPDCVEKLIMT 1296
Equus caballus                  KYSNADVNRLRVHAMTQHSVQPMLRCPLCQDMLNNKIHLQLHLTHLHSVAPDCVEKLIMT 1296
Trichechus manatus latirostris  KYSNADINRLRVHAMTQHSVQPMLRCPLCQDMLNNKIHLQLHLTHLHSVAPDCVEKLIMT 1292
Sus scrofa                      KYSNADVNRLRVHAMTQHSVQPMLRCPLCQDMLNNKIHLQLHLTHLHSVAPDCVEKLIMT 1302
Felis catus                     KYSNADVNRLRVHAMTQHSVQPMLRCPLCQDMLNNKIHLQLHLTHLHSVAPDCVEKLIMT 1295
Gorilla gorilla                 KYSNADVNRLRVHAMTQHSVQPMLRCPLCQDMLNNKIHLQLHLTHLHSVAPDCVEKLIMT 1290
Ursus arctos horribilis         KYSNADVNRLRVHAMTQHSVQPMLRCPLCQDMLNNKIHLQLHLTHLHSVAPDCVEKLIMT 1295
Loxodonta africana              KYSNADVNRLRVHAMTQHSVQPMLRCPLCQDMLNNKIHLQLHLTHLHSVAPDCVEKLIMT 1293
Bos taurus                      KYSNADVNRLRVHAMTQHSVQPMLRCPLCQDMLNNKIHLQLHLTHLHSVAPDCVEKLIMT 1306
Myotis lucifugus                KYSNADVNRLRVHAMTQHSVQPMLRCPLCQDMLNNKIHLQLHLTHLHSVAPDCVEKLIMT 1285
Rattus norvegicus               KYSNADVNRLRVHAMTQHSVQPMLRCPLCQDMLNNKIHLQLHLTHLHSVAPDCVEKLIMT 1287
Mus musculus                    KYSNADVNRLRVHAMTQHSVQPLLRCPLCQDMLNNKIHLQLHLTHLHSVAPDCVEKLIMT 1298
                                ******:***************:*************************************
```

C

p.Q2564R variant

```
Homo sapiens                    LPPQLIPYQCDQCKLAFPSFEHWQEHQLHFLSAQNQFIHPQFLDRSLDMPFMLFDPSNP 2582
Pan troglodytes                 LPPQLIPYQCDQCKLAFPSFEHWQEHQLHFLSAQNQFIHPQFLDRSLDMPFMLFDPSNP 2578
Vulpes vulpes                   LPPQLIPYQCDQCKLAFPSFEHWQEHQLHFLSAQNQFIHPQFLDRSLDMPFMLFDPSNP 2598
Canis lupus familiaris          LPPQLIPYQCDQCKLAFPSFEHWQEHQLHFLSAQNQFIHPQFLDRSLDMPFMLFDPSNP 2599
Equus caballus                  LPPQLIPYQCDQCKLAFPSFEHWQEHQLHFLSAQNQFIHPQFLDRSLDMPFMLFDPSNP 2592
Trichechus manatus latirostris  LPPQLIPYQCDQCKLAFPSFEHWQEHQLHFLSAQNQFIHPQFLDRSLDMPFMLFDPSNP 2590
Sus scrofa                      LPPQLIPYQCDQCKLAFPSFEHWQEHQLHFLSAQNQFIHPQFLDRSLDMPFMLFDPSNP 2602
Felis catus                     LPPQLIPYQCDQCKLAFPSFEHWQEHQLHFLSAQNQFIHPQFLDRSLDMPFMLFDPSNP 2580
Gorilla gorilla                 LPPQLIPYQCDQCKLAFPSFEHWQEHQLHFLSAQNQFIHPQFLDRSLDMPFMLFDPSNP 2581
Ursus arctos horribilis         LPPQLIPYQCDQCKLAFPSFEHWQEHQLHFLSAQNQFIHPQFLDRSLDMPFMLFDPSNP 2581
Loxodonta africana              LPPQLIPYQCDQCKLAFPSFEHWQEHQLHFLSAQNQFIHPQFLDRSLDMPFMLFDPSNP 2581
Bos taurus                      LPPQLIPYQCDQCKLAFPSFEHWQEHQLHFLSAQNQFIHPQFLDRSLDMPFMLFDPSNP 2592
Myotis lucifugus                LPPQLIPYQCDQCKLAFPSFEHWQEHQLHFLSAQNQFIHPQFLDRSLDMPFMLFDPSNP 2565
Rattus norvegicus               LPPQLIPYQCDQCKLAFPSFEHWQEHQLHFLSAQNQFIHPQFLDRSLDMPFMLFDPSNP 2582
Mus musculus                    LPPQLIPYQCDQCKLAFPSFEHWQEHQLHFLSAQNQFIHPQFLDRSLDMPFMLFDPSNP 2591
                                ***********************************************************
```

Predicted zinc-finger

Supplemental Figure S1

Supplemental Figure S2. *ZFHX3* mRNA expression represented as cycle to threshold (ΔCt) values and measured in HL-1 cells transfected with the cDNA encoding WT or mutated Zfhx3.

ZFHX3 mRNA expression

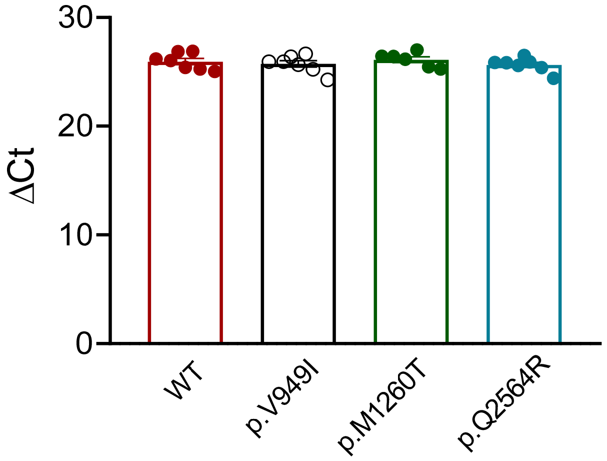

Supplemental Figure S2

Supplemental Figure S3. Sequence of the minimal promoters of human *SCN5A, SCN1B,* and *TBX5* genes with the AT motifs marked using bold and black font.

Human *SCN5A* minimal promoter

TACCATTGTGCAGATTTCTGTGGCATCACTAAGTGTGTCTCCAGGAAAGTGTGGTTCGAGTGAGTGTGTGTGTCTCT
GTGTCAGTGTGTCAGTGGATGTGTGTCTAAGTCATGTGGGTGTCTAAGTTTGTCAGAGTGTCTTCCTCAGTGTCAGT
GTGTG**TCAATGAT**GTGTCTGTCTGTGTCTGTGTGGGTGTGTGGGGGTGTGTATCTGTCTCAGGGTGCATGTGTGTGT
CTGTGTCAGTGCAGGTATGCCTATGGCAGCGTGTGTCTGTCTCGGCATGAGTGTCAGTGTATGCCCGTGTCCACGTG
GGTGGGTGGGTGTCTGGTAGCCTTCCCAAGTCAGCAAGGTTGCGTGTGTGTGTG**TGTATACT**CTGGCGGGTGCTGGT
GTGTATGCCAGTG**TTTGTTAAT**GTGAGCCTGTCCGCGTCCGCGTGGGTGGCCATCTGTGGTGAAGCGTCGCCGGGTC
GCCGTGTGTGTACCCCCGCCTATGTCTGTCTGTCCGCGGCCGCGTGTGCGGCTGTCTGTGGCTGTGAGCCCCCGGGT
CAGTGTGGGAGTGTGCGCCCCGGCGGGCGCTGGTGTGTCCCCCCTCCGAGCGGGCGGAGCACCACGTGCGGAGCCCT
GGGCGCGTCGCTCTGGGGCGGAGCCAGCCCGGGGCCCCAAGCCCCAGGCCGAACCCAGGCGGGGCCCGCCCCGACCC
CGCCCCCGACCCCGCCCCCAGCCCGAGCCCGCGCCGCCTGCCCAGCCCGGGAGCCCGAACAGAGCCGCGGAGCCGAG
ACGGCGGCGGCGCCCGTAGGATGCAGGGATCGCTCCCCCGGGGCCGCTGAGCCTGCGCCCAGTGCCCCGAGCCCCGC
GCCGAGCCGAGTCCGCGCCAAGCAGCAGCCGCCCACCCCGGGGCCCGGCCGGGGGACCAGCAGGTGAGCGAGTGCCC
CCGCGCCCGGCAGCCCTGGCCCGGCCGGAGCCCCCTCCGCCTGCCGCCGCCC**AACTTT**CCTCCCCGAGGGC

Human *SCN1B* minimal promoter

CTCAGGGGAGATAACTTGGAGGTTCGCCCTCTGGAACTCCTGAGGATGACCTCC**ATCATTT**GAAGTCGGAAACCCGG
CCCCCAAACTGCGCTCTAAGTCCTCCTGTGACTCTGGGGGTGACCTTTCCAGACTCCCTGGGAAGGTGGGGGGGGG
GGGTGGTCCTCAGAGCCCCAGCGGGGTCGGAGACACACAGACCACCCTCCCCGTTCCCAGAAGTCGCGGTCCCTGCG
CCACCCGGAGAGGCCGGTGCAGCGGGCGCCTCCCGGGCCGCTGCGGCAAAGGCTGGGCGGCCGCGCCTTCCCCCCGC
GGTGATTCATCCCGCCCCCTCCCTCTCCTCCCTCGTCCCTCCCTAGGCCGCCGCCGCCGCCGCCGCCGCCGCCGCTG
CAGTGCGCAGGAGACCGCGGTCCGCGCCCGAGCGCGCCCGAGCCGGAGCGGGACCGGGGTCGGTGCACCTAGCGGAT
GTGCCCGGCTGCGCGCGCCAGCGCAGCAGCCCGAGCAGCGGCCGCCGCCCGCGCGGCGGGGATGCCCGGACGCCGGG
CCCCGGGGCTGGGCCCCCGGCGGTAACCGGAGCGGGGGGGCCGCGCCCCCCCTCCTCCCCCCTCGCCGGTCCCAGAG
CCGCAGCTGCTGCGCCCGCGCGCTCCCGGGGAC**ATTCTAA**CCGCCGCCAGGTCCCGCCGCCTCTCGCCCCGC**TATTA**
ATACCGGCGGCCCGGGAGGGGGGCGCAGCACGCGCCGCGCAGCCATGGGGAGGCTGCTGGCCTTAGTGGTCGGCGCG
GCACTGGGTGAGTGCGCGGGGGGCGCGCGCGGCCGGGGGGCACCGCGGGGGCACTGGCGGGGCGGCGGGAGTGGCGC
TCGGGACACGGGGCAGCCGCGCGAGGGCCACCCCCGGCCCATCCCCGGGCCCCGCCAAGTCAGCTTCAGAAGT

Human *TBX5* minimal promoter

AGTATGATGGGGGTACAGGAGGTAAGGATGGGAAACAGAACAGGACTCCTGAC**ATTTA**CTCCAAGCACCAATATTCG
GTGTCCCAATCTGGGTCTGCTCAGACTGAGACCTACTGACTGTGTTTGTGC**TTTTATT**GACCTCTTCAACTCACCC**T**
AAAAAAAAAAAATACATCTCAGAGAGGGAAAGGGCAAGGAGAGGAGACCAGTGGAGATGAAAACCCTAGCCAGTCCC
CAGTGACTG**TTT**G**ATTATTTTAATAAAA**CTGGGCACTGCCTTGTGTACACCTGGAGCAGGGGACGTCTGGTGGCCCC
ACTGGGTGGGGGGTGAGGGCCCGCAGCG**ATTTCTTA**GCATCTTTGATCTCGGCCCCCATCCCAATGCACCTTCACCC
TGCCTTCACCCCAGAGTCGTTGAGAGTAGGGGTGATGAGTAGGGGGTGGAGGGGAGATGTCAGGAAGGCGAGCGCCG
GCCAGGCGGGGTCAGGCAGCTCTCCTTCTCGAGGTCAGCGTTGGAGAGAATGTCTGCAAGGCTGCGGAGGCCCGCGG
TGTGTTTGTGTGTGTGCGTCCAGACTCGGTTCTCTGCACCGCCAGCGTCACTGAGATTACTTCCCGATTAGAAGCCG
ACCGCG**TTTGAAATGATTT**GTGCAGGAGTTTTTGCAGCCACCGCTTGCTCAGAGAAGCAGAGATGGATGGAGGTTGG
GAAAGGGGTAGAGAGGAGGGAG**TTATT**GCAGGTCTGTGTTGAGAGTCGTATTGTGATTTGAGTGTTCGGGAAATCTA
GTGG**AAATTT**GGGGTGGGGGGAAGGGAGGACGGGAGGGTGGGAGGGAGAGAGAAGGGGGAGGGCGACAGAGTGCAGT
GGGAGCTAGTTGGATAGGCGATTTCAGTACTTTGTGAGCATCGAGGCAACCCAACGTCACTGTGCTCAGCTGAGTTG
GCTTGTATTTCAGAGAGAGAGAGAGAGGGAGAGAGAGTGAGAGAGACTGACTCTTACCTCGAATCCGGGAAC**TTTAA**
TCCTGAAAGCTGCGCTCAGAAAGGACTTCGACCATTCAC

Supplemental Figure S3

Supplemental Tables

Supplemental Table S1. Predicted risk of missense *ZFHX3* variants as calculated by the Combined Annotation Dependent Depletion score.

ZFHX3 **variants**	**CADD Score**
**Identified in the present paper**	
**p.V949I**	3.19
**p.M1260T**	2.02
**p.Q2564R**	2.92
**AF-associated variants** [1]	
**p.E460Q**	2.58
**p.V777A**	0.87
**p.M1476I**	1.47
**p.S3513G**	0.51

Supplemental Table S2. List of genes selected for the bioinformatics analysis performed after Whole Exome Sequencing.

No.	Gene	Protein name	Location
1	*ABCC9*	ATP-binding cassette, sub-family C (CFTR/MRP), member 9 (SUR2)	12p12.1
2	*ACTN2*	Actinin Alpha 2	1q43
3	*AGXT2*	Alanine--Glyoxylate Aminotransferase 2	5p13.2
4	*AKAP5*	A-Kinase Anchoring Protein 5	14q23.3
5	*AKAP9*	A kinase (PRKA) anchor protein (yotiao) 9	7q21-q22
6	*ALPK3*	Alpha Kinase 3	15q25.3
7	*ANK2*	Ankyrin 2	4q25-q27
8	*ANK3*	Ankyrin 3	10q21.2
9	*ANKRD1*	Ankyrin Repeat Domain 1	10q23.31
10	*ATF4*	Activating Transcription Factor 4	22q13.1
11	*ATP1B1*	ATPase Na^+/K^+ Transporting Subunit Beta 1	1q24.2
12	*B3GNT7*	UDP-GlcNAc:BetaGal Beta-1,3-N-Acetylglucosaminyltransferase 7	2q37.1
13	*BIN1*	Bridging Integrator 1	2q14.3
14	*C9ORF3*	Chromosome 9 open reading frame 3 (aminopeptidase O)	9q22.32
15	*CACNA1C*	Calcium channel, voltage-dependent, L type, alpha 1C subunit	12p13.3
16	*CACNA1D*	Calcium channel, voltage-dependent, L type, alpha 1D subunit	3p14.3
17	*CACNA1G*	Calcium channel, voltage-dependent, T type, alpha 1G subunit	17q22
18	*CACNA1H*	Calcium channel, voltage-dependent, T type, alpha 1H subunit	16p13.3
19	*CACNA1I*	Calcium channel, voltage-dependent, T type, alpha 1I subunit	22q13.1
20	*CACNA2D1*	Calcium channel, voltage-dependent, alpha 2/delta subunit 1	7q21-q22
21	*CACNA2D2*	Calcium channel, voltage-dependent, alpha 2/delta subunit 2	3p21.31
22	*CACNB2*	Calcium channel, voltage-dependent, beta 2 subunit	10p12
23	*CALM1*	Calmodulin 1	14q32.11
24	*CALM2*	Calmodulin 2	2p21
25	*CALM3*	Calmodulin 3	19q13.32
26	*CAMK2D*	Calcium/Calmodulin Dependent Protein Kinase II Delta	4q26
27	*CAND2*	Cullin Associated And Neddylation Dissociated 2	3p25.2
28	*CASP3*	Caspase 3	4q35.1
29	*CASQ2*	Calsequestrin 2 (cardiac muscle)	1p13.3-p11
30	*CAV1*	Caveolin 1	7q31.2
31	*CAV2*	Caveolin 2	7q31.2
32	*CAV3*	Caveolin 3	3p25
33	*CDH2*	Cadherin 2	18q12.1
34	*CREB1*	cAMP Responsive Element Binding Protein 1	2q33.3
35	*CREB5*	cAMP Responsive Element Binding Protein 5	7p15.1-p14.3
36	*CREM*	cAMP Responsive Element Modulator	10p11.21
37	*CTNNA3*	Catenin Alpha 3	10q21.3
38	*CUX2*	Cut Like Homeobox 2	12q24.11-q24.12
39	*DES*	Desmin	2q35
40	*DLG1*	Discs, large homolog 1 (SAP97)	3q29
41	*DPP6*	Dipeptidyl-peptidase 6	7q36.2
42	*DTNA*	Dystrobrevin Alpha	18q12.1
43	*ECRG4*	Esophageal Cancer Related Gene 4 Protein Augurin Precursor	2q12.2
44	*EMD*	Emerin	Xq28
45	*ENPP4*	Ectonucleotide Pyrophosphatase/Phosphodiesterase 4	6p21.1
46	*EPAS1*	Endothelial PAS Domain Protein 1	2p21
47	*ETV1*	ETS Variant Transcription Factor 1	7p21.2
48	*FBN1*	Fibrillin 1	15q21.1
49	*FBN2*	Fibrillin 2	5q23.3
50	*FBXO32*	F-Box Protein 32	8q24.13
51	*FGF12*	Fibroblast growth factor 12	3q28-q29

52	*FGF13*	Fibroblast growth factor 13	Xq26.3
53	*FLNA*	Filamin A	Xq28
54	*FLNB*	Filamin B	3p14.3
55	*FLNC*	Filamin C	7q32.1
56	*GALNT1*	Polypeptide N-Acetylgalactosaminyltransferase 1	18q12.2
57	*GATA4*	GATA binding protein 4	8p23.1-p22
58	*GATA5*	GATA binding protein 5	20q13.33
59	*GATA6*	GATA binding protein 6	18q11.2
60	*GJA1*	Gap junction protein, alpha 1, 43kDa (Cx43)	6q22-q23
61	*GJA5*	Gap junction protein, alpha 5, 40kDa (Cx40)	1q21.1
62	*GPC5*	Glypican 5	13q32
63	*GPD1L*	Glycerol-3-phosphate dehydrogenase 1-like	3p22.3
64	*HAND2*	Heart And Neural Crest Derivatives Expressed 2	4q34.1
65	*HCN1*	Hyperpolarization activated cyclic nucleotide-gated potassium channel 1	5p12
66	*HCN2*	Hyperpolarization activated cyclic nucleotide-gated potassium channel 2	19p13
67	*HCN3*	Hyperpolarization activated cyclic nucleotide-gated potassium channel 3	1q21.2
68	*HCN4*	Hyperpolarization activated cyclic nucleotide-gated potassium channel 4	15q24.1
69	*HEY2*	Hes Related Family BHLH Transcription Factor With YRPW Motif 2	6q22.31
70	*IRX5*	Iroquois homeobox 5	16q12.2
71	*ISL1*	ISL LIM Homeobox 1	5q11.1
72	*JPH2*	Junctophilin 2	20q13.12
73	*KCNA4*	Potassium voltage-gated channel, shaker-related subfamily, member 4	11p14
74	*KCNA5*	Potassium voltage-gated channel, shaker-related subfamily, member 5	12p13
75	*KCNA7*	Potassium voltage-gated channel, shaker-related subfamily, member 7	19q13.3
76	*KCNAB1*	Potassium Voltage-Gated Channel Subfamily A Member Regulatory Beta Subunit 1	3q25.31
77	*KCNB1*	Potassium voltage-gated channel, Shab-related subfamily, member 1	20q13.2
78	*KCNB2*	Potassium voltage-gated channel, Shab-related subfamily, member 2	8q13.2
79	*KCND2*	Potassium voltage-gated channel, Shal-related subfamily, member 2	7q31
80	*KCND3*	Potassium voltage-gated channel, Shal-related subfamily, member 3	1p13.2
81	*KCNE1*	Potassium voltage-gated channel, Isk-related family, member 1	21q22.1-q22.2
82	*KCNE1L*	Potassium voltage-gated channel, Isk-related family, member 1-like	Xq22.3
83	*KCNE2*	Potassium voltage-gated channel, Isk-related family, member 2	21q22.1
84	*KCNE3*	Potassium voltage-gated channel, Isk-related family, member 3	11q13.4
85	*KCNE4*	Potassium voltage-gated channel, Isk-related family, member 4	2q36.1
86	*KCNH2*	Potassium voltage-gated channel, subfamily H (eag-related), member 2	7q36.1
87	*KCNIP2*	Kv channel interacting protein 2	10q24.32
88	*KCNJ11*	Potassium inwardly-rectifying channel, subfamily J, member 11 (Kir6.2)	11p15.1
89	*KCNJ12*	Potassium inwardly-rectifying channel, subfamily J, member 12 (Kir2.2)	17p11.1
90	*KCNJ2*	Potassium inwardly-rectifying channel, subfamily J, member 2 (Kir2.1)	17q24.3

91	*KCNJ3*	Potassium inwardly-rectifying channel, subfamily J, member 3 (Kir3.1)	2q24.1
92	*KCNJ4*	Potassium inwardly-rectifying channel, subfamily J, member 4 (Kir2.3)	22q13.1
93	*KCNJ5*	Potassium inwardly-rectifying channel, subfamily J, member 5 (Kir3.4)	11q24
94	*KCNJ8*	Potassium inwardly-rectifying channel, subfamily J, member 8 (Kir6.1)	12p12.1
95	*KCNK2*	Potassium Two Pore Domain Channel Subfamily K Member 2	1q41
96	*KCNK3*	Potassium Two Pore Domain Channel Subfamily K Member 3	2p23.3
97	*KCNK17*	Potassium Two Pore Domain Channel Subfamily K Member 17	6p21.2
98	*KCNN3*	Potassium intermediate/small conductance calcium-activated channel, subfamily N, member 3	1q21.3
99	*KCNQ1*	Potassium voltage-gated channel, KQT-like subfamily, member 1	11p15.5
100	*KCNV1*	Potassium channel, subfamily V, member 1	8q23.2
101	*KLF10*	Kruppel Like Factor 10	8q22.3
102	*KLF12*	Kruppel Like Factor 12	13q22.1
103	*LDB3*	LIM Domain Binding 3	10q23.2
104	*LGALS3*	Galectin 3	14q22.3
105	*LRIT3*	Leucine Rich Repeat, Ig-Like And Transmembrane Domains 3	4q25
106	*MEF2D*	Myocyte Enhancer Factor 2D	1q22
107	*MEIS1*	Meis Homeobox 1	2p14
108	*MYH6*	Myosin Heavy Chain 6	14q11.2
109	*MYOCD*	Myocardin	17p12
110	*NCS1*	Neuronal calcium sensor 1	9q34.11
111	*NDRG4*	NDRG Family Member 4	16q21
112	*NEBL*	Nebulette	10p12.31
113	*NEDD4*	NEDD4 E3 Ubiquitin Protein Ligase	15q21.3
114	*NEURL1*	Neuralized E3 Ubiquitin Protein Ligase 1	10q24.33
115	*NFIA*	Nuclear Factor I A	1p31.3
116	*NKX2-5*	NK2 Homeobox 5	5q34
117	*NKX2-6*	NK2 Homeobox 6	8p21.2
118	*NOS1AP*	Nitric Oxide Synthase 1 Adaptor Protein	1q23.3
119	*NODAL*	Nodal Growth Differentiation Factor	10q22.1
120	*NOTCH1*	Notch Receptor 1	9q34.3
121	*NPR1*	NPPA receptor A	1q21.3
122	*PBX1*	PBX Homeobox 1	1q23.3
123	*PITX2*	Paired-like homeodomain 2	4q25
124	*PKP1*	Plakophilin 1	1q32.1
125	*PKP2*	Plakophilin 2	12p11.21
126	*PKP3*	Plakophilin 3	11p15.5
127	*PKP4*	Plakophilin 4	2q24.1
128	*PLN*	Phospholamban	6q22.1
129	*PPFIA4*	PTPRF Interacting Protein Alpha 4	1q32.1
130	*PRMT3*	Protein arginine methyltransferase 3	11p15.1
131	*PRMT5*	Protein arginine methyltransferase 5	14q11.2
132	*PRRX1*	Paired Related Homeobox 1	1q24.2
133	*RANGRF*	RAN guanine nucleotide release factor (MOG1)	17p13
134	*RBM20*	RNA Binding Motif Protein 20	10q25.2
135	*REM2*	RRAD And GEM Like GTPase 2	14q11.2
136	*RFX4*	Regulatory Factor X4	12q23.3
137	*RHOA*	Ras Homolog Family Member A	3p21.31
138	*RNF207*	Ring Finger Protein 207	1p36.31
139	*ROCK1*	Rho-Associated, Coiled-Coil-Containing Protein Kinase 1	18q11.1
140	*ROCK2*	Rho-Associated, Coiled-Coil-Containing Protein Kinase 2	2p25.1
141	*RRAD*	Ras Related Glycolysis Inhibitor And Calcium Channel Regulator	16q22.1
142	*SCN10A*	Sodium channel, voltage-gated, type X, alpha subunit	3p22.2
143	*SCN1B*	Sodium channel, voltage-gated, type I, beta subunit	19q13.12
144	*SCN2B*	Sodium channel, voltage-gated, type II, beta subunit	11q22-qter

145	*SCN3B*	Sodium channel, voltage-gated, type III, beta subunit	11q24.1
146	*SCN4B*	Sodium channel, voltage-gated, type IV, beta subunit	11q23.3
147	*SCN5A*	Sodium channel, voltage-gated, type V, alpha subunit	3p21
148	*SCN8A*	Sodium channel, voltage gated, type VIII, alpha subunit	12q13.1
149	*SEMA3A*	Semaphorin 3[a]	7q21.11
150	*SGK1*	Serum/Glucocorticoid Regulated Kinase 1	6q23.2
151	*SHOX2*	Short Stature Homeobox 2	3q25.32
152	*SLC6A4*	Solute Carrier Family 6 Member 4	17q11.2
153	*SLC8A1*	Solute Carrier Family 8 Member A1	2p22.1
154	*SLC28A1*	Solute Carrier Family 28 Member 1	15q25.3
155	*SLC35F1*	Solute Carrier Family 35 Member F1	6q22.2-q22.31
156	*SLMAP*	Sarcolemma associated protein	3p21.2-p14.3
157	*SNTA1*	Syntrophin, alpha 1 (dystrophin-associated protein A1, 59kDa, acidic component)	20q11.2
158	*SOX5*	SRY-Box Transcription Factor 5	12p12.1
159	*SOX8*	SRY-Box Transcription Factor 8	16p13.3
160	*SPHK1*	Sphingosine Kinase 1	17q25.1
161	*SPON1*	Spondin 1	11p15.2
162	*SPTBN2*	Spectrin Beta, Non-Erythrocytic 2	11q13.2
163	*SPTBN4*	Spectrin Beta, Non-Erythrocytic 4	19q13.2
164	*SSBP3*	Single Stranded DNA Binding Protein 3	1p32.3
165	*STRN*	Striatin, calmodulin binding protein	2p22.2
166	*SYNE2*	Spectrin Repeat Containing Nuclear Envelope Protein 2	14q23.2
167	*SYNPO2L*	Synaptopodin 2 Like	10q22.2
168	*TBX20*	T-box 20	7p14.3
169	*TBX3*	T-box 3	12q24.1
170	*TBX5*	T-box 5	12q24.1
171	*TBX18*	T-box 18	6q14.3
172	*TCAP*	Titin-Cap	17q12
173	*TMEM43*	Transmembrane Protein 43	3p25.1
174	*TRDN*	Triadin	TRDN
175	*TRPC3*	Transient Receptor Potential Cation Channel Subfamily C Member 3	4q27
176	*TRPM4*	Transient receptor potential cation channel, subfamily M, member 4	19q13.3
177	*VCL*	Vinculin	10q22.2
178	*WNT4*	Wnt Family Member 4	1p36.12
179	*WNT8A*	Wnt Family Member 8A	5q31.2
180	*ZBTB17*	Zinc Finger And BTB Domain Containing 17	1p36.13
181	*ZFHX3*	Zinc Finger Homeobox 3	16q22.2-q22.3
182	*ZNFX1*	Zinc Finger NFX1-Type Containing 1	20q13.13

Supplemental Material and Methods

1. *Study approval.*

The study was approved by the Investigation Committees of the University Hospitals La Paz, 12 de Octubre y Puerta de Hierro (Madrid, Spain) in the context of the ITACA Consortium and conforms to the principles outlined in the Declaration of Helsinki. Each participant gave written informed consent.

2. *Clinical evaluation.*

The probands and their relatives were evaluated by the Arrhythmia Units of the University Hospitals La Paz, 12 de Octubre y Puerta de Hierro. Complete clinical evaluation, including ECG, transthoracic echocardiogram, and exercise test were performed in all of them.

3. *Whole-exome sequencing.*

Whole-exome sequencing was performed at NIMgenetics (Madrid, Spain) following described procedures (1). DNA was extracted from whole blood by using the DNeasy Blood & Tissue kit (Qiagen, Hilden, Germany) following the manufacturer's protocol. The Qubit™ 2.0 and NanoDrop devices (Thermo Fisher Scientific Inc., Waltham, MA, USA) were used to determine DNA concentration and purity. The AmpliSeq™ Exome panel (Thermo Fisher Scientific Inc.) was used for library preparation. This technique captures >97% of consensus coding sequences (>19,000 genes, >198,000 exons, >85% of alterations responsible for genetic diseases) and adjacent splicing regions (5 bp). The panel is approximately 33 Mb in size and comprises a total of 293,903 amplicons. Libraries were quantified by qPCR and subsequently prepared and enriched using the Ion Chef™ system providing a high uniformity of coverage. Library sequencing at a mean

depth of coverage of >100X was performed using the Ion Proton (Thermo Fisher Scientific Inc.) sequencing platform, covering >92% of amplicons with at least 20X. The sequences obtained were aligned against the hg19 GRCh37 reference genome using the Torrent Mapping Alignment Program software (GitHUB Inc., San Francisco, CA, USA). The sequences, aligned and filtered according to specific quality criteria, were analyzed with the Torrent Variant Caller tool to identify nucleotide variations with respect to the reference genome. Variant annotation was performed using the latest available version of Ion Reporter™ (Thermo Fisher Scientific Inc.). The analysis aimed to identify rare variants, single nucleotide polymorphisms (SNPs) and indels (excluding synonymous variants) located in the exons and splicing junctions of the genes, which were detected in over 40% of reads. Out of the whole exome, we analyzed the coding and splicing regions of 182 genes (Supplementary Table S2) including: a) genes already associated to primary arrhythmogenic syndromes, b) constitutive proteins of cardiac ion channels, c) proteins that participate in cardiac channelosomes, and d) proteins whose involvement in the modulation of ion channel activity has been demonstrated (functional studies) or suggested (Genome-Wide-Association studies). Most of the genes associated to inherited structural cardiomyopathy diseases were excluded. Genes underlying catecholaminergic polymorphic ventricular tachycardia were also excluded, since patients with this condition were not genotyped. We evaluated the list of variants identified against database information on previously described variants (http://www.ncbi.nlm.nih.gov/SNP/, http://www.1000genomes.org, http://gnomad.broadinstitute.org, and http://evs.gs.washington.edu/EVS). Firstly, we excluded variants located in introns (except those in suspected splicing sites) or intergenic regions. We also removed synonymous variants and non-synonymous variants with occurrences >1 in our local database. In order to understand the possible biological functions of the variants selected,

we estimated the functional effect of the genomic variations classified as pathogenic following the current recommendations of the American College of Medical Genetics and Genomics and the Association for Molecular Pathology (2, 3). Variant pathogenicity was graded according to its presence in a previously associated or candidate gene, the *in silico* predicted impact on the protein using widely used software (Polyphen2, SIFT, PROVEAN, Mutation Taster, Mutation Assessor and Likelihood Ratio Test-LRT), the degree of conservation of the affected residue measured by multiple ortholog alignment using Alamut software (http:// www.interactive-biosoftware.com) and its presence in public databases such as dbSNP, genome aggregation (https:// gnomad.broadinstitute.org/), and 1000 genomes. Potentially pathogenic variants were confirmed by Sanger sequencing.

4. *Sanger sequencing.*

Variants in the *ZFHX3* gene found in the probands were confirmed by Sanger and identified in their relatives by the same method (3). PCR products were purified using illustra ExoProStar 1-Step (GE Healthcare Life Sciences, Piscataway, NJ, USA) and the analysis was performed by direct sequencing using the Applied Biosystems ABI Prism 3730 DNA sequencer (Secugen S.L. and NIMgenetics, Madrid, Spain). The results were compared with the reference sequence from hg19 by means of Chromas Lite Software (http://technelysium.com.au).

5. Access to public human cardiac RNA-seq data.

The GTEx project collects and analyzes 54 non-diseased post mortem tissue sites across nearly 1000 individuals. Samples were used for molecular assays including Whole Genome Sequencing, Whole Exome Sequencing, and RNA-Seq (GTEx Consortium.,

2013; Melé et al., 2015). GTEx RNA-seq data from human atria (n=297) and ventricles (n=303) and the resulting transcripts Per Million (TPM) values for *ZFHX3*, *TBX5*, *PITX2*, and *NKX25* were accessed through the Human Protein Atlas database (https://www.proteinatlas.org/) [RSEMv1.2.22 (v7)] (4).

6. *Site-Directed Mutagenesis.*

Human *ZFHX3* cDNA subcloned in pCMV6-AC-GFP was purchased on Origene (catalog number: RG 223762). The p.V949I and p.M1260T variants in *ZFHX3* were introduced using *Platinum Superfii II* polymerase (ThermoFisher Scientific Inc.). The p.Q2564R variant was introduced using the Q5® Site-Directed Mutagenesis Kit (NewEnglandBiolabs, Ipswich, MA, USA). The three mutations were confirmed by direct DNA sequencing as previously described (3-8).

7. *HL-1 cell culture and transfection.*

HL-1 cells (Sigma Aldrich, St Louis, MO, USA), a cardiomyocyte cell line from murine origin, were cultured in 60-mm gelatin/fibronectin-coated dishes at 37ºC in an 5% CO_2 atmosphere, as previously described (3, 9). HL-1 cells (mycoplasma free) were transfected with Zfhx3 WT, p.V949I, p.M1260T or p.Q2564R (2 μg) by using Lipofectamine 2000 (Invitrogen, Carlsbad, CA, USA), according to the manufacturer´s instructions (3, 9). Forty-eight h after transfection, cells were removed from the dish by trypsinization (1%, for 5 min at room temperature) and used for electrophysiological or expression experiments.

8. *Patch-clamp recordings.*

Currents were recorded at room temperature (21-23ºC) by means of the whole-cell patch-clamp technique using an Axopatch-200B patch clamp amplifier (Molecular Devices, San José, CA, USA)(3-15). Recording pipettes were pulled from 1.0 mm o.d. borosilicate capillary tubes (GD1, Narishige Co., Ltd, Tokyo, Japan) using a programmable patch micropipette puller (Model P-2000 Brown-Flaming, Sutter Instruments Co., Novato, CA, USA) and were heat-polished with a microforge (Model MF-83, Narishige). Micropipette resistance was kept below 1.5 MΩ when filled with the internal solution and immersed in the external solution. In all the experiments, series resistance was compensated manually by using the series resistance compensation unit of the Axopatch amplifier, and ≥80% compensation was achieved. The remaining access resistance after compensation and cell capacitance were 1.6±0.7 MΩ and 20.9±2.3 pF (n=41) in untransfected HL-1 cells. Neither WT nor mutated Zfhx3 forms significantly modified these values. On the other hand, mean maximum I_{Na} amplitude at -20 mV was -76.7±6.8 pA/pF (n=24). Therefore, under our experimental conditions no significant voltage errors (<5 mV) due to series resistance were expected with the micropipettes used. To minimize the contribution of time-dependent shifts of channel availability during I_{Na} recordings, all data were collected 5-10 min after establishing the whole-cell configuration. Under these conditions current amplitudes and voltage dependence of activation and inactivation were stable during the time of recordings (3-6, 8, 12, 15). Current recordings were sampled at 50 kHz, filtered at half the sampling frequency and stored on the hard disk of a computer for subsequent analysis. To minimize the influence of the expression variability, each construct was tested in a large number of cells obtained from at least 3 different HL-1 batches. In all cases the expression of WT or mutated Zfhx3 was identified by the green fluorescent signal under fluorescent microscopy (Nikon Eclipse TE2000S, Nikon, Tokyo, Japan). **Solutions.**

The external solution contained (mM): NaCl 100, CsCl 50, $MgCl_2$ 1.5, $CaCl_2$ 1, HEPES 5, glucose 5, and nifedipine (1 μM) (pH=7.35 with CsOH). Recording pipettes were filled with an internal solution containing (mM): NaF 10, CsF 110, CsCl 20, HEPES 10, and EGTA 10 (pH=7.35 with CsOH).

Pulse protocols and analysis (3-6, 8, 12)**.**

To construct the current-voltage relationships, 50-ms pulses in 5 mV increments from -120 mV to potentials between -90 and +20 mV were applied. To analyze the recovery from inactivation of I_{Na}, two 50-ms pulses (P1 and P2) from –120 to -20 mV were applied at increasing coupling intervals (0.05-500 ms). A monoexponential function was fitted to the data to measure the reactivation kinetics. To construct the inactivation-availability curves, the I_{Na} was recorded by applying 500-ms pulses from -120 mV to potentials between -140 and -20 mV in 10 mV increments followed by a test pulse to −20 mV. Inactivation curves were constructed by plotting the current amplitude recorded with the test pulse as a function of the membrane potential of the preceding pulse. Conductance-voltage curves were constructed by plotting the normalized conductance as a function of the membrane potential. The conductance was estimated for each experiment by the equation:

$$G=I/(V_m-E_{rev})$$

where G is the conductance at the test potential V_m, I represents the peak maximum current at V_m, and E_{rev} is the reversal potential. To determine the E_{rev}, I_{Na} density-voltage relationships obtained in each experiment were fitted to a function of the form:

$$I=(V_m-E_{rev})*G_{max}*(1+\exp[V_m-V_h]/k)^{-1}$$

where I is the peak current elicited at the test potential V_m, G_{max} is the maximum conductance, and *k* is the slope factor.

A Boltzmann function was fitted to activation/conductance-voltage and inactivation curves to obtain the midpoint (V_h) and the slope (*k*) values of the curves. In order to describe the time course of I_{Na} decay, a biexponential analysis was used as an operational approach, fitting the inactivating phase of the peak current traces with an equation of the form:

$$y=C+A_f*\exp(-t/\tau_f)+A_s*\exp(-t/\tau_s)$$

where τ_f and τ_s are the fast and slow time constants, whereas A_f and A_s are the amplitudes of each component of the exponential, and C is the baseline value. The activation kinetics was described by fitting a monoexponential function to the activating phase of the peak current traces yielding the corresponding τ_{act}.

I_{NaL} was recorded by applying 500-ms pulses from -120 to -20 mV, measured by the end of protocol, and represented as the percentage of the peak I_{Na} amplitude. Data were analyzed using pCLAMP software (Molecular Devices).

9. *Analysis of the mRNA expression (Reverse Transcription Quantitative PCR-rt-qPCR).*

RNA was extracted from HL-1 cells transfected or not with WT and mutant Zfhx3 using the *NucleoSpin RNA kit* (Macherey-Nagel, Allentown, PA, USA) according to the manufacturer's instructions (4). RNA was quantified in Nano-Drop 2000 (ThermoFisher Scientific Inc.). Reverse transcription (RT)-qPCR was performed by the Genomic Facility at UCM (https://www.ucm.es/gyp/genomica-3) using the High Capacity cDNA Reverse Transcription kit (ThermoFisher Scientific Inc.). The resulting cDNA template was subjected to real-time quantitative PCR (qPCR) using a Taqman-based gene assay, TaqMan Fast Universal PCR Master Mix (ThermoFisher Scientific Inc.) and the 7900HT Fast Real-Time PCR System (ThermoFisher Scientific Inc.). Gene expression analysis was performed using TaqMan Gene Expression Assays (ThermoFisher Scientific Inc.):

Hs00199344_m1 (*ZFHX3*), Mm00451979_m1 (*Scn5a*), Mm00441210_m1 (*Scn1b*), Mm00459584_m1 (*Nedd4l*), Mm00803518_m1 (*Tbx5*), and Mm01309813_s1 (*Nkx25*). The *Hprt1* (Mm_03024075_m1) rRNA endogenous control was used as the normalization gene. Each sample was run in triplicate and non-template control to test for contamination of assay reagents was also included in the plate. Moreover, three different controls aimed at detecting genomic DNA contamination in the RNA sample or during the RT or qPCR reactions were always included: a RT mixture without reverse transcriptase, a RT mixture including the enzyme but no RNA, and a negative control (reaction mixture without cDNA template). The data were collected and analyzed using One-Step Software (ThermoFisher Scientific Inc.). The obtained cycle to threshold (Ct) values were normalized to the *Hprt1* RNA. The Ct values are based on a log scale and were transformed to delta Ct (ΔCt) values by subtracting the value corresponding to the gene of interest from that of *Hprt1*. We attempted to determine the expression of *Pitx2* mRNA in HL-1 cells by using two different TaqMan Gene Expression probes (Mm00440826_m1 o Mm01316994_m1). Unfortunately, we were not able to accurately detect its expression in rt-qPCR experiments.

10. *Western blot analysis.*

Detection of Zfhx3, Nav1.5, Nedd4-2, and Tbx5 proteins was carried out in HL-1 cells transfected or not with WT Zfhx3 by Western blot following previously described procedures (3-6, 8, 9, 12). HL-1 cells were collected in RIPA buffer containing 50 mM Tris·HCl (pH=7.5), 150 mM NaCl, 1% Nonidet P-40, 0.1% SDS, 0.5% sodium deoxycholate, and 1 mM phenylmethylsulfonyl fluoride (PMSF) and protease inhibitor cocktail (Sigma).

In extracts from HL-1 cells nuclei and cell debris were removed by centrifugation at 14.000 rpm for 20 min at 4ºC. The total protein amount of the extracts was calculated with the bicinchoninic acid method (Pierce, San Antonio, TX, USA). Samples were run on 4-15% Mini-PROTEAN TGX™ stain-free gels (Bio-Rad Laboratories Inc., Hercules, CA, USA) and transferred to nitrocellulose membranes. Nonspecific binding sites were blocked with 5% nonfat dried milk in PBS with Tween-20 (0.05%) for 1 hour at room temperature. Membranes were then incubated with rabbit polyclonal anti-Zfhx3 primary antibody (1:1000; PD011 MBL International, Woburn, MA, USA), rabbit polyclonal anti-Nav1.5 (1:1000; S0819 Sigma), rabbit monoclonal anti-Nedd4-2 (1:2500; MA5-32294 ThermoFisher Scientific Inc.), and rabbit polyclonal anti-Tbx5 (1:500; 42-6500 ThermoFisher Scientific Inc.) primary antibodies overnight at 4ºC. These antibodies bind to residues 3405-3549 (C-terminal domain) of mouse Zfhx3, residues 493-511 (intracelular linker between DI and DII) of human Nav1.5, residues 931-980 of human Nedd4-2 and an internal region of human Tbx5, respectively. Afterwards, samples were incubated for 1 hour with peroxidase-conjugated goat anti-rabbit (1:10,000; 111-035-144 Jackson Immunoresearch, Westgrove, PA, USA), goat anti-mouse (1:10,000; 115-035-146 Jackson Immunoresearch) or rabbit anti-goat (1:4000; AP106P Sigma) secondary antibody. Membranes were washed three times with PBS-Tween after adding primary and secondary antibodies. Protein expression was detected by chemiluminescence (SuperSignal™ West Femto Maximum Sensitivity Substrate, ThermoFisher Scientific Inc.) and visualized using the Chemidoc MP System and Image Lab 5.2.1. software (Bio-Rad Laboratories). The total protein was used as a loading control by means of stain-free gels (Bio-Rad Laboratories) and ImageLab software (3-6, 8, 9, 12). Expression of the proteins in the immunoblot was then normalized to the total protein. Measurement of the protein expression of Pitx2c in HL-1 cells was attempted by using the anti-Pitx2 primary

antibody (1:400, AV35634 Sigma). However, we did not detect it probably due to a very low basal expression of the protein in these cells.

For Zfhx3 silencing, HL-1 cells were transfected with ON-TARGETplus mouse Zfhx3 siRNA SMARTpool (L-042881-01-0005, Horizon, Dublin, Ireland) (5 nM) or with siRNA Universal Negative Control (scrambled, Sigma) by using Lipofectamine 2000 (Invitrogen) (3-6, 8), according to manufacturer instructions. The target sequences of the siRNA duplexes against mouse Zfhx3 (NM_007496.2) are the following: J-042881-09, 5’-[illegible]GGAGGAGUCGUUUA-3’; J-042881-10, 5’-GGAUAAACCAAACGAGUUA-3’; J-042881-11, 5’-CGGAAGAAGUUAGCGGAUA-3’ and J-042881-12, 5’-GCUUUGUGCUCUACGGCAA-3’. Overexpression and silencing was confirmed by Western blot using the anti-Zfhx3 primary antibody mentioned above. Electrophysiological and Western blot analyses were performed 24 h after siRNA transfection.

11. *Luciferase gene expression reporter assays.*

For luciferase reporter assays (3, 9), HL-1 cells were seeded in 96-well plates and cultured as mentioned above. Cells were transfected with 100 ng of pLightSwitch_Prom luciferase expression reporter vectors carrying the minimal promoters (≈1000 bp) of human *SCN5A, SCN1B, TBX5*, *PITX2*, or *NKX25* genes (Active Motif, Carlsbad, CA, USA). In these experiments, cells were cotransfected with 100 ng of an empty vector, Zfhx3 WT or p.V949I, p.M1260T or p.Q2564R variants. Forty-eight h after transfection luciferase assays were conducted using the *Renilla*-Glo® Luciferase Assay System (Promega, Madison, WI, USA) and a Berthold Luminometer (Berthold Technologies, Badwildbad, Germany). All reporter assays were performed in triplicate.

12. *Statistical analysis.*

Results are expressed as mean±SEM. Statistical analysis was performed using GraphPad Prism 8 (GraphPad Software, San Diego, CA, USA). To compare data from ≥3 experimental groups one-way ANOVA followed by Tukey's test was used. Unpaired two-sided t-test was chosen when comparing data from 2 experimental groups. In small-size samples ($n<5$), statistical significance was confirmed by using nonparametric tests (two-sided Wilcoxon's test). To take into account repeated sample assessments, data were analyzed with multilevel mixed-effects models. Normality assumption was verified using the Shapiro–Wilk test. Variance was comparable between groups throughout the manuscript. We chose the appropriate tests according to the data distributions. A value of $P<0.05$ was considered significant. For the different groups of experiments sample size was chosen empirically according to previous experience in the calculation of experimental variability. No statistical method was used to predetermine sample size. No particular procedure was followed for randomization/allocation of the respective experimental groups.

Supplementary References

1. Pytel V, Matías-Guiu JA, Torre-Fuentes L, Montero-Escribano P, Maietta P, Botet J, et al. Exonic variants of genes related to the vitamin D signaling pathway in the families of familial multiple sclerosis using whole-exome next generation sequencing. Brain Behav. 2019;9(4):e01272.
2. Richards S, Aziz N, Bale S, Bick D, Das S, Gastier-Foster J, et al. Standards and guidelines for the interpretation of sequence variants: a joint consensus recommendation of the American College of Medical Genetics and Genomics and the Association for Molecular Pathology. Genet Med. 2015;17(5):405-24.
3. Caballero R, Utrilla RG, Amoros I, Matamoros M, Perez-Hernandez M, Tinaquero D, et al. Tbx20 controls the expression of the KCNH2 gene and of hERG channels. Proc Natl Acad Sci U S A. 2017;114(3):E416-E25.
4. Nieto-Marin P, Tinaquero D, Utrilla RG, Cebrian J, Gonzalez-Guerra A, Crespo-Garcia T, et al. Tbx5 variants disrupt Nav1.5 function differently in patients diagnosed with Brugada or Long QT Syndrome. Cardiovasc Res. 2021.
5. Tinaquero D, Crespo-Garcia T, Utrilla RG, Nieto-Marin P, Gonzalez-Guerra A, Rubio-Alarcon M, et al. The p.P888L SAP97 polymorphism increases the transient outward current (Ito,f) and abbreviates the action potential duration and the QT interval. Sci Rep. 2020;10(1):10707.
6. Pérez-Hernández M, Matamoros M, Alfayate S, Nieto-Marín P, Utrilla RG, Tinaquero D, et al. Brugada syndrome trafficking–defective Nav1. 5 channels can trap cardiac Kir2. 1/2.2 channels. JCI insight. 2018;3(18).
7. Nieto-Marin P, Jimenez-Jaimez J, Tinaquero D, Alfayate S, Utrilla RG, Rodriguez Vazquez Del Rey MDM, et al. Digenic Heterozigosity in SCN5A and CACNA1C Explains the Variable Expressivity of the Long QT Phenotype in a Spanish Family. Rev Esp Cardiol (Engl Ed). 2019;72(4):324-32.
8. Matamoros M, Perez-Hernandez M, Guerrero-Serna G, Amoros I, Barana A, Nunez M, et al. Nav1.5 N-terminal domain binding to alpha1-syntrophin increases membrane density of human Kir2.1, Kir2.2 and Nav1.5 channels. Cardiovasc Res. 2016;110(2):279-90.
9. Pérez-Hernández M, Matamoros M, Barana A, Amorós I, Gómez R, Núñez M, et al. Pitx2c increases in atrial myocytes from chronic atrial fibrillation patients enhancing I Ks and decreasing I Ca, L. Cardiovasc Res. 2016;109(3):431-41.
10. Vaquero M, Caballero R, Gomez R, Nunez L, Tamargo J, Delpon E. Effects of atorvastatin and simvastatin on atrial plateau currents. J Mol Cell Cardiol. 2007;42(5):931-45.
11. Gómez R, Núñez L, Vaquero M, Amorós I, Barana A, de Prada T, et al. Nitric oxide inhibits Kv4. 3 and human cardiac transient outward potassium current (I to1). Cardiovasc Res. 2008;80(3):375-84.
12. Utrilla RG, Nieto-Marín P, Alfayate S, Tinaquero D, Matamoros M, Pérez-Hernández M, et al. Kir2. 1-Nav1. 5 channel complexes are differently regulated than Kir2. 1 and Nav1. 5 channels alone. Front Physiol. 2017;8:903.
13. Caballero R, Dolz-Gaiton P, Gomez R, Amoros I, Barana A, Gonzalez de la Fuente M, et al. Flecainide increases Kir2.1 currents by interacting with cysteine 311, decreasing the polyamine-induced rectification. Proc Natl Acad Sci U S A. 2010;107(35):15631-6.
14. Caballero R, de la Fuente MG, Gómez R, Barana A, Amorós I, Dolz-Gaitón P, et al. In humans, chronic atrial fibrillation decreases the transient outward current and ultrarapid component of the delayed rectifier current differentially on each atria and

increases the slow component of the delayed rectifier current in both. J Am Coll Cardiol. 2010;55(21):2346-54.
15. Dolz-Gaiton P, Nunez M, Nunez L, Barana A, Amoros I, Matamoros M, et al. Functional characterization of a novel frameshift mutation in the C-terminus of the Nav1.5 channel underlying a Brugada syndrome with variable expression in a Spanish family. PLoS One. 2013;8(11):e81493.
